# General Anesthesia in Early Childhood Significantly Reduces Asthma Incidence and Clinical Visits: A Nationwide Population-Based Cohort Study

**DOI:** 10.3390/children10040626

**Published:** 2023-03-27

**Authors:** Ya-Ling Yang, Jung-Chan Chang, Shu-Chen Ho, Chien-Ning Yeh, Ho-Chang Kuo

**Affiliations:** 1 Department of Anesthesiology, Kaohsiung Chang Gung Memorial Hospital, Kaohsiung City 833, Taiwan; inr453@cgmh.org.tw; 2 Department of Data Science and Analytics, I-Shou University, Kaohsiung 840, Taiwan; 3 Department of Public Health, College of Health Sciences, Kaohsiung Medical University, Kaohsiung 807, Taiwan; 4 Department of Pediatrics, Kaohsiung Chang Gung Memorial Hospital and Chang Gung University College of Medicine, Kaohsiung 833, Taiwan; 5 Kawasaki Disease Center, Kaohsiung Chang Gung Memorial Hospital, Kaohsiung 833, Taiwan

**Keywords:** general anesthesia, asthma, incidence, outcome

## Abstract

Few studies have focused on the consequence of exposure to general anesthesia (GA) in children’s early life with the risk of asthma and disease outcomes. The present study examines the correlation between exposure to GA under three years old and the subsequent course of asthma in a nationwide population-based cohort study. Our cases were acquired from Taiwan’s National Health Insurance Research Database (NHIRD). Children under three years old with either GA exposure or not during in-patient treatment from 1997 to 2008 were included. The study group was age- and sex-matched with a ratio of 1:2 to create the control group for comparison. The cohort included 2261 cases with GA and 4522 cases without GA as a control group. The incidence of asthma onset was significantly reduced in patients with GA exposure under 3 three years old (hazard ratio 0.64 (95% confidence interval 0.57~0.72), *p* < 0.001). In addition, regardless of whether the asthmatic clinical visits were before or after GA exposure, asthma onset patients before GA exposure have significantly fewer clinical visits than those without GA exposure (both *p <* 0.001, respectively). Using the Kaplan–Meier method, we also demonstrated that GA exposure was associated with favorable clinical visits in patients with asthma, whether their asthma was onset before GA (*p* = 0.0102) or after GA exposure (*p =* 0.0418) compared to non-GA-exposed controls. In the present study, we demonstrated that children with early GA exposure under three years old were at a reduced risk of developing asthma compared to the general population. Furthermore, we first reported that GA exposure significantly reduced clinical visits in patients with asthma regardless of whether their asthma onset was before or after GA exposure. It is indicated that GA exposure at a younger age could have potential clinical benefits for asthma than non-GA-exposed controls.

## 1. Introduction 

The rising worldwide incidences of asthma represent a vital healthcare issue for children [1,2,3], and its associated clinical symptoms may worsen life quality [4] and bring an economic burden [5,6]. Mounting pieces of evidence of various factors, including pathogenic bacteria colonization in the airway during the early life of children [7], exposure to smoke [8,9], O2 therapy [10], indoor allergen [11,12], air pollution [13,14], and climate change [15,16], have triggered the growing prevalence of asthma in children. Meanwhile, according to the hygiene hypothesis, improving hygienic environment standards and decreasing the chances of infections can subsequently increase the incidence of asthma in children [17]. However, Hallit et al. have demonstrated that neither home cleaning nor personal cleanliness was correlated with asthma in preschool children [18]. Therefore, understanding how asthma develops and resolves through infancy and childhood is essential for the pediatrician. Fortunately, global asthma incidence and lethality have been reported to decline from 1990 to 2019 [1].

Except for necessary surgery, the most common etiologies of received GA during early childhood are minor surgery, including inguinal hernia [19], redundant prepuce, phimosis [20], and hydrocele [21]. In addition, there is a steady growth of the widespread use of GA in children, such as dental treatment [22,23], MRI image study [24], or removal of a foreign body in the external auditory canal, nose, or esophagus [25,26]. The reasons for using GA in these procedures are not only that it can reduce procedure time [22] and anxiety [27] but also the possibility of constant subsiding postoperative pain [28]. However, the safety of GA has become an essential health issue of interest to the public and government agencies [29]. Notably, we had reported that children exposed to GA early, before age three, had a small association with an increased risk of developmental delay after that [30]. Furthermore, in an animal study exposed to postnatal GA, adult mice have chronically exacerbated fear behavior, associated with an 11% reduction of the periaqueductal gray matte compared to the non-GA group [31]. In addition, it is shown that multiple sevoflurane exposures induced remarkable learning ability impairment in younger but not adult mice involved in the function of Egr2, a critical protein for age-dependent vulnerability to sevoflurane-induced cognitive deficits [32]. Younger children exposed to GA were found to have almost no differences in intelligence compared with the unexposed group. Notably, some studies have found more behavioral problems, particularly attention-deficit hyperactivity disorder, after multiple exposures in children with GA exposure [33]. On the other hand, our group previously reported that children had a lower risk for allergic diseases, including allergic rhinitis, asthma, and atopic dermatitis, with intubated or general mask GA exposure before one year old compared to the general population without GA exposure [34].

Asthma is a chronic inflammatory process of the lower respiratory airways and a Type 2-high (Th2) disease [35]. Some researchers believe that GA may be involved in immune function by affecting the balance of Th1 and Th2 in favor of Th1 responses [36] or stress responses caused by surgery [37]. GA may advance proinflammatory Th1 and decrease Th2 responses and thus potentially prevent the risk of allergic diseases. In addition, one prior study has consistently found increased pro-inflammatory IL-6 cytokine in postoperative patients after GA [38]. However, studies on GA and asthma in children are minimal and insufficient, so the relationship between GA and the later development of asthma and the severity of asthma remains to be determined. Therefore, this nationwide population-based case-control study aimed to investigate the relationship between GA exposure and subsequent asthma and the clinical course of asthma after exposure to GA in children under three years old. All the participants in this study were followed for at least five years.

## 2. Methods

Our study data were gained from Taiwan’s National Health Insurance Research Database with diagnoses based on all the International Classification of Diseases diagnostic codes, Ninth Revision, and Clinical Modification (ICD-9-CM) format [34]. Taiwan’s National Health Insurance project was launched on 1 March 1995 and provides universal health insurance for 99% of the 23 million residents [39]. Many studies have delineated the details of a representative database of 1,000,000 subjects randomly sampling from the National Health Insurance Research Database. It includes the patients’ background characteristics such as gender, date of birth, disease diagnosis, medical information, medicine prescriptions, clinical visits, or hospitalizations, which are provided to the public for research purposes [30,40,41]. Additionally, there were no statistically significant differences in age, gender, and healthcare costs between the samples and all enrollees [39,40]. Meanwhile, the patient's identification was encrypted to protect their privacy.

**Figure 1:**
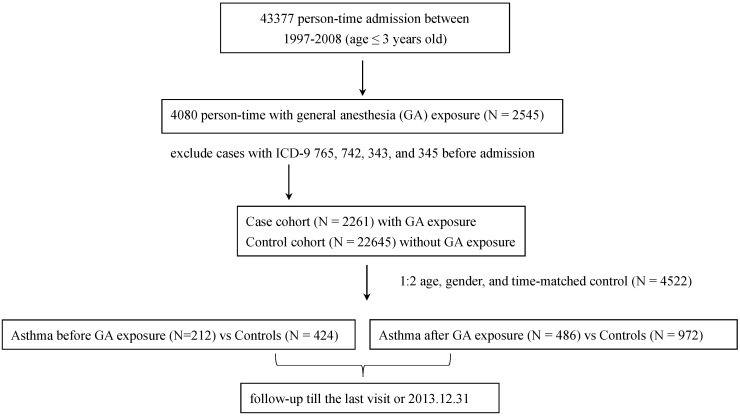
Algorithm for study design and patient selection. The study cohort included 43,377 children under the age of three years who were hospitalized from 1997 to 2008. Among them, 4080 person-times were exposed to GA. After exclusion cases using ICD-9 codes 765 (disorders related to short gestation and low birthweight), 742 (other congenital anomalies of the nervous system), 343 (infantile cerebral palsy), and 345 (epilepsy and recurrent seizures) before admission, we ultimately included 2261 subjects in the study cohort, while 4522 children (in a 1:2 ratio, matched by age, sex, and time) made up the control cohort. The observation period started on the index date and ended on the last clinical visit on December 31, 2013. The length of follow-up time was calculated for each patient’s last clinical visit. To avoid patients with overdiagnosis of asthma (ICD-9 493), the enrolled patients have had at least two outpatient visits (within 28 days) or one inpatient visit. In addition, we investigated asthma development and clinical visits after GA exposure.

Our study subjects were children under three years old hospitalized between 1997 and 2008, without or with GA exposure. We excluded those cases with any premature birth (ICD-9-CM 765), other congenital system abnormalities (ICD-9-CM 742), epilepsy (ICD-9-CM 345), and pediatric cerebral palsy (ICD-9-CM 343). According to the management these patients received, they were divided into study and control groups: the study group was hospitalized patients with exposure, and the control group was a non-GA-exposed cohort. Therefore, it was a 1:2 ratio of a case-control study. Then, we performed an age, gender, and hospital admission during the same period-matched study (±7 days). The observation period started on the index date and ended on the last clinical visit on 31 December 2013. The length of follow-up time was calculated for each patient’s last clinical visit. To avoid patients with overdiagnosis of asthma (ICD-9 493), the enrolled patients have had at least two outpatient visits (within 28 days) or one inpatient visit. The flowchart of data collection in this study is demonstrated in Figure 1. Since this study used the National Health Insurance Research Database, it was exempted from full review by the Institutional Review Board (No. 102-0364B on 3 April 2013) of Chang Gung Memorial Hospital.

## 3. Statistical Analysis

The time of follow-up person-years for each subject was investigated from either the last clinical visit or 31 December 2013. The incidence rate was calculated by dividing the number of cases of patients by the number of follow-up person-years. We used Cox proportional hazard regression models adjusted for all potential confounders to estimate the relative risks associated with GA exposure or non. We calculated the hazard ratios (HRs) and their 95% confidence intervals (CIs) using non-GA patients as a reference. We adopted the SAS statistical software package (SAS Institute Inc., Cary, NC, USA; version 9.3) for analysis. We used statistical methods of Student’s t-test (two-tailed), χ^2^ test (two-tailed), and Kaplan–Meier graphs with the log-rank test for data analysis. Any *p*-value of <0.05 was represented as statistical significance.

## 4. Results

### 4.1. General Anesthesia in Early Childhood Significantly Reduces Asthma Incidence

The study cohort included 43,377 children under the age of three years who were hospitalized from 1997 to 2008. The flowchart of this research is shown in Figure 1. Among them, 4080 person-times were exposed to GA. After, exclusion cases using ICD-9 codes 765 (disorders related to short gestation and low birthweight), 742 (other congenital anomalies of the nervous system), 343 (infantile cerebral palsy), and 345 (epilepsy and recurrent seizures) before admission were used. We enrolled 2261 subjects in the study group, while a 1:2 ratio, matched by age, sex, and time, with 4522 subjects as the control cohort. We first investigated asthma development after GA exposure. The significant finding was that incidence of asthma in the GA-exposed group was lower than that in the non-GA group (23.78% vs. 34.28 %, hazard ratio 0.64 (95% confidence interval 0.57~0.72), *p <* 0.001).

### 4.2. General Anesthesia in Early Childhood Significantly Reduces Clinical Asthma Visits

To compare the clinical outcome of patients following GA exposure, we divided the patients with the onset of asthma before and after GA exposure. In literature, the most common etiologies of receiving pediatric surgery are redundant prepuce, inguinal hernia, hydrocele, and phimosis [42], which are male-predominant and consistent with our results of the GA group in Table 1. Table 1 shows no difference in age, gender, years of follow-up, or clinical visits in asthmatic patients’ onset before GA exposure compared to non-GA-exposed patients. Cumulative clinical visits indicate the summation of outpatient and inpatient visits. Of particular note, GA-exposed patients had fewer outpatient visits (11.33 ± 17.90 vs. 17.47 ± 27.60, *p =* 0.0008) and cumulative clinical visits (11.55 ± 18.22 vs. 17.92 ± 28.30, *p =* 0.0007) when compared to non-GA-exposed patients. However, we found no difference in asthmatic patients’ onset after GA exposure compared to non-GA-exposed controls in clinical visits (Table 2). Furthermore, we divided the frequency of clinical visits into low, medium, and high according to the non-GA-exposed group. As shown in Table 3 of asthmatic patients’ onset before GA exposure, the GA-exposed group has a lower rate of high (>14) outpatient and cumulative clinical visits (0.40, 95%, CI 0.21~0.73; 0.39, 95% CI 0.21~0.73, respectively) compared to non-GA-exposed controls. In patients with asthma onset after GA exposure, the GA-exposed group also demonstrated a lower rate of medium (5~≤ 13) (0.47, 95% CI 0.32~0.69, respectively) and high (>13) cumulative clinical visits (0.15, 95% CI 0.10~0.23) than non-GA-exposed controls (Table 4). It indicated that for asthma onset before and after GA, the GA-exposed group had lower clinical visits than non-GA-exposed controls.

### 4.3. Kaplan–Meier Plot of the Cumulative Incidence of Asthmatic Patients following GA Exposure

**Figure 2:**
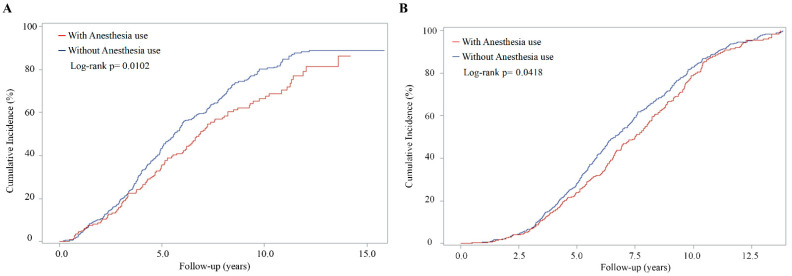
Kaplan–Meier plot of the cumulative incidence of asthmatic patients following general anesthesia (GA) exposure. It is showed that patients with GA exposure (red line) were associated with favorable clinical visits with asthma than the non-GA exposure group (blue line), regardless of whether their asthma onset was (**A**) before GA (*p =* 0.0102) or (**B**) after GA exposure (*p =* 0.0418).

Using the Kaplan–Meier method, we also confirmed that GA exposure was associated with favorable clinical visits in patients with asthma, regardless of whether their asthma onset was before GA (Figure 2A, *p =* 0.0102) or after GA exposure (Figure 2B, *p =* 0.0418). Taken altogether, we showed that GA exposure (red line) significantly reduced clinical visits in patients with asthma compared to non-GA-exposed controls (blue line).

## 5. Discussion

In this study, we further proved evidence that children exposed to GA before the age of three had a reduced risk of subsequently developing asthma in the nationwide population-based case-control study (23.78% vs. 34.28 %, hazard ratio 0.64 (95% confidence interval 0.57~0.72), *p <* 0.001). Meanwhile, we are the first to report that GA exposure significantly reduced later clinical visits in patients regardless of whether their asthma onset was before or after GA exposure. It is indicated that no matter whether asthma onset is before or after GA, GA exposure could have the potential clinical benefit over non-GA-exposed controls.

Most asthmatic children with acute exacerbation can be effectively treated with β2-adrenergic agonists and corticosteroids [43]. However, status asthmaticus is an intractable attack refractory to standard treatment that can lead to progressive respiratory failure [44]. Therefore, in refractory status asthmaticus, volatile anesthetics are also used for pediatric patients who do not respond well to conventional therapy [45,46,47]. The proposed mechanisms for volatile anesthetics include activating the β-adrenergic receptors, inhibiting acetylcholine and histamine release, and directly depressing airway reflexes and inducting bronchial smooth muscle relaxation that reverses the underlying airway bronchoconstriction [46,48]. Halothane, isoflurane, and enflurane are effectual bronchodilators and can be used in patients with status asthmaticus; however, sevoflurane has revealed controversial results in asthmatic patients [49,50]. Furthermore, halothane, isoflurane, and sevoflurane are asthmatic surgical patients’ best induction and maintenance agents [50].

Our previous study reported that children receiving GA exposure before one year of age reduced the risk of developing allergic diseases, including asthma, allergic rhinitis, and atopic dermatitis, by approximately 25–40% [34]. However, one study of children’s exposure to GA did not demonstrate either an increased or decreased risk of atopic dermatitis (2.3%) compared to the non-GA-exposed group (2.2%) when followed up for two years after cohort entry [51]. The authors stated that board-certified dermatologists only diagnosed the participants in that study with subsequent atopic dermatitis. Therefore, the incidence of atopic dermatitis may be underestimated due to the many patients who follow up with pediatricians, especially with allergic-immunologic and rheumatologic subspecialties, instead of board-certified dermatologists. With the more restrictive enrollment criteria of asthma who have had at least two outpatient visits (within 28 days) or one inpatient visit in this study, we further confirmed a 36% reduction in the risk of asthma development in the group of GA exposure compared to non-GA-exposed subjects in this study. Although no evidence has linked GA with a decrease in asthma later in life, various theories may explain the possible mechanisms for the relationship between GA exposure and asthma in children [52]. Theoretically, it is possible that GA promotes inflammatory Th1 responses and decreases Th2 immunity which may be protective against the development of asthma.

Sevoflurane has numerous advantages in children, including blood solubility, pleasant odor, and less bronchospasm [53,54,55]. Because of the high prevalence of airway hyper-reactivity and an increased risk of bronchospasm, desflurane was suggested to be avoided in asthmatic pediatric anesthesia [56]. Furthermore, multiple sevoflurane exposures do not interfere with the T-cell receptor repertoire in baby monkeys’ thymus [57]. Consistently, Chutipongtanate et al. showed that desflurane could induce higher peripheral blood Tregs increment than sevoflurane after 24-h exposure [58]. Furthermore, sevoflurane could increase the CD4 + lymphocytes in the spleen in mice, augment antibody-producing capacity following the antigenic challenge, and increase the number of peripheral blood leukocytes after one or repeated exposures [59]. In addition, sevoflurane potentiates host-defense mechanisms of bactericidal and anti-inflammatory reactions in endotoxemia [60]. Furthermore, Wang et al. showed that sevoflurane could mitigate allergic airway inflammation induced by ovalbumin in mice by inhibiting Th2 responses [61]. Koksoy et al. also uncovered that GA, rather than spinal anesthesia, altered the balance of Th1 and Th2 in favor of Th1 responses after surgery [9]. Notably, a neonatal animal study showed that sevoflurane influenced genetic methylation [62] and histone acetylation [63], which may explain reducing allergic disease after GA exposure in the earlier stage of life. Taken together, the possible explanation is that GA can promote inflammatory Th1 reactions and decrease Th2 responses, which may result in a decline in the risk of asthma after GA exposure in an earlier stage of children. However, it is also believed that surgery itself may affect the regulatory balance of postoperative immune responses [64,65]. Interferon-gamma, an important Th1 cytokine, was increased 24 h after surgery in patients anesthetized by halothane and isoflurane [66]. However, further studies in other countries are needed to support the conclusion that exposure to GA and its long-term effects affect the subsequent development of asthma in children.

Our current study has some limitations that should be mentioned. First, the results gained from this study were based on a nationwide population-based cohort study, and additional countries or global studies are still needed to affirm the beneficial effect of GA on asthma. Second, many confounding factors may be associated with asthma development; therefore, selecting good controls for comparison is difficult. However, we chose age-, gender-, and time-matched controls for comparison to reduce confounding factors. Meanwhile, an additional GA-exposed group who has anesthesia only was assured for comparison for diagnostic sedation or the removal of foreign bodies, such as from the nose, eyes, ear, esophagus, etc., without surgery or with minor surgery (such as redundant prepuce, inguinal hernia, dental treatment). Third, frequent respiratory tract infection is a predisposing factor to the development of asthma later. Thus, a more extensive database for excluding respiratory tract infections before GA exposure or not is needed for comparison. On the other hand, we will know what kind of disease admission the risk factor for the development of asthma is. Fourth, we need to find out how long the protective effect in the development of asthma is, and the topic thus still warrants further study.

## 6. Conclusions

Asthma represents a significant public health issue of interest for children. Except for necessary surgery, children have an increased risk of GA exposure during early childhood for minor surgery, procedures, or examinations. However, current studies on GA and asthma in children are minimal and insufficient, so the relationship between GA and the later development of asthma and the severity of asthma remains to be determined. This nationwide population-based cohort study reported that, compared with the general population, children exposed to GA before three years had a reduced risk of asthma. Moreover, we are the first to report that GA exposure significantly reduced clinical visits in patients with asthma. It is indicated that GA exposure at a younger age could have potential clinical benefits for asthma than non-GA-exposed controls.

## Figures and Tables

**Table 1:** Characteristics of the patients with asthma onset before general anesthesia (GA) exposure and unexposed subjects.

Variables	Patients with GA Exposure		Patients Without GA Exposure		*p*-Value
	n	%	n	%	
	n = 212		n = 424		
Age (means ± SD)	2.07 ± 0.60		2.01 ± 0.54		0.2234
Follow-up (years, mean ± SD)	5.36 ± 3.62		5.06 ±3.63		0.3289
Gender					
Female	71	33.49	142	33.49	
Male	141	66.51	282	66.51	
Clinical visits before GA					
Outpatient visits (No., mean ± SD)	4.19 ± 5.77		4.74 ± 6.50		0.2995
Cumulative clinical visits (No., mean ± SD)	4.47 ± 5.93		5.31 ± 6.90		0.1108
Clinical visits after GA					
Outpatient visits (No., mean ± SD)	11.33 ± 17.90		17.47 ± 27.60		0.0008
Cumulative clinical visits (No., mean ± SD)	11.55 ± 18.22		17.92 ± 28.30		0.0007

Cumulative clinical visits indicate the combined total of both outpatient and inpatient visits.

**Table 2:** Characteristics of the patients with asthma onset after general anesthesia (GA) exposure and unexposed subjects.

Variables	Patients with GA Exposure		Patients without GA Exposure		*p*-Value
	n	%	n	%	
After asthma	n = 486		n = 972		
Age (mean ± SD)	1.83 ± 0.59		1.83 ± 0.59		0.8077
Follow-up (years, mean ± SD)	5.59 ± 2.93		5.24 ± 2.99		0.0373
Gender					
Female	144	29.63	288	29.63	
Male	342	70.37	684	70.37	
Clinical visits					
Outpatient visits (No., mean ± SD)	12.49 ± 15.61		13.84 ± 17.34		0.1369
Cumulative clinical visits. (No., mean ± SD)	12.70 ± 15.84		14.10 ± 17.51		0.1245

Cumulative clinical visits indicate the combined total of both outpatient and inpatient visits.

**Table 3:** Following clinical visit numbers in patients with asthma onset before general anesthesia (GA) exposure.

	No. of Patients	No. of Person-Years	No. of Patients with Anesthesia Use	Incident Rate (per 10,000 Person-Years)	Crude HR (95% CI)	Adjusted HR * (95% CI)
Gender						
Female	213	1078.11	71	658.56	1.00	
Male	423	2205.35	141	639.35	0.98 (0.73~1.30)	
No. of outpatient visits						
0~≤3	246	1282.02	99	772.22	1.00	1.00
3~≤14	205	805.74	62	769.48	1.00 (0.57~1.75)	1.04 (0.59~1.84)
>14	185	1195.70	51	426.53	0.39 (0.21~0.71)	0.40 (0.21~0.73)
Cumulative No. of clinical visits						
0~≤3	242	1271.92	98	770.49	1.00	1.00
3~≤14	204	800.95	63	786.57	1.03 (0.89~1.81)	1.07 (0.60~1.90)
>14	190	1210.59	51	421.28	0.38 (0.21~0.71)	0.39 (0.21~0.73)

* Adjusted for age and gender. Cumulative clinical visits indicate the combined total of both outpatient and inpatient visits.

**Table 4:** Following clinical visit numbers in patients with asthma onset after general anesthesia (GA) exposure.

	No. of Patients	No. of Person-Years	No. of Patients with Anesthesia Use	Incident Rate (per 10,000 Person-Years)	Crude HR (95% CI)	Adjusted HR * (95% CI)
Gender						
Female	432	2215.26	144	650.04	1.00	
Male	1026	5605.02	342	610.17	0.89 (0.73~1.08)	
No. of outpatient visits						
0~≤4	517	2024.61	191	943.39	1.00	1.00
4~≤17	605	3324.76	192	577.49	0.32 (0.22~0.47)	0.33 (0.22~0.48)
>17	336	2470.91	103	416.85	0.12 (0.07~0.20)	0.12 (0.07~0.20)
Cumulative No. of clinical visits						
0~≤5	613	2481.10	216	870.58	1.00	1.00
5~≤13	400	2129.65	134	629.21	0.46 (0.31~0.68)	0.47 (0.32~0.69)
>13	445	3209.53	136	423.74	0.15 (0.10~0.24)	0.15 (0.10~0.23)

* Adjusted for age and gender. Cumulative clinical visits indicate the combined total of both outpatient and inpatient visits.

## Data Availability

The datasets generated and analyzed during the current study are not publicly available due to strict ethical regulations of information privacy, but are available from the corresponding author Ho-Chang Kuo on reasonable request.

## References

[1] Cao Y., Chen S., Chen X., Zou W., Liu Z., Wu Y., Hu S. (2022). Global trends in the incidence and mortality of asthma from 1990 to 2019: An age-period-cohort analysis using the global burden of disease study 2019. Front. Public Health.

[2] Batra M., Dharmage S.C., Newbigin E., Tang M., Abramson M.J., Erbas B., Vicendese D. (2022). Grass pollen exposure is associated with higher readmission rates for pediatric asthma. Pediatr. Allergy Immunol..

[3] De Keyser H.H., Chipps B., Dinakar C. (2021). Biologics for Asthma and Allergic Skin Diseases in Children. Pediatrics.

[4] Rattu A., Khaleva E., Brightling C., Dahlén S.E., Bossios A., Fleming L., Roberts G. (2022). Identifying and appraising outcome measures for severe asthma: A systematic review. Eur. Respir. J..

[5] Zuberbier T., Lotvall J., Simoens S., Subramanian S.V., Church M.K. (2014). Economic burden of inadequate management of allergic diseases in the European Union: A GA(2) LEN review. Allergy.

[6] Foronda C.L., Kelley C.N., Nadeau C., Prather S.L., Lewis-Pierre L., Sarik D.A., Muheriwa S.R. (2020). Psychological and Socioeconomic Burdens Faced by Family Caregivers of Children With Asthma: An Integrative Review. J. Pediatr. Health Care.

[7] Bisgaard H., Chawes B., Stokholm J., Mikkelsen M., Schoos A.M., Bonnelykke K. (2023). 25 Years of translational research in the Copenhagen Prospective Studies on Asthma in Childhood (COPSAC). J. Allergy Clin. Immunol..

[8] Klain A., Dinardo G., Salvatori A., Indolfi C., Contieri M., Brindisi G., Decimo F., Zicari A.M., Miraglia Del Giudice M. (2022). An Overview on the Primary Factors That Contribute to Non-Allergic Asthma in Children. J. Clin. Med..

[9] Koksoy S., Sahin Z., Karsli B. (2013). Comparison of the effects of desflurane and bupivacaine on Th1 and Th2 responses. Clin. Lab..

[10] Kim A., Lim G., Oh I., Kim Y., Lee T., Lee J. (2018). Perinatal factors and the development of childhood asthma. Ann. Allergy Asthma Immunol..

[11] Maciag M.C., Phipatanakul W. (2022). Update on indoor allergens and their impact on pediatric asthma. Ann. Allergy Asthma Immunol..

[12] Grant T., Lilley T., McCormack M.C., Rathouz P.J., Peng R., Keet C.A., Rule A., Davis M., Balcer-Whaley S., Newman M. (2023). Indoor environmental exposures and obstructive lung disease phenotypes among children with asthma living in poor urban neighborhoods. J. Allergy Clin. Immunol..

[13] Cushing A.M., Khan M.A., Kysh L., Brakefield W.S., Ammar N., Liberman D.B., Wilson J., Shaban-Nejad A., Espinoza J. (2022). Geospatial data in pediatric asthma in the United States: A scoping review protocol. JBI Evid. Synth..

[14] Moore L.E., Oliveira A., Zhang R., Behjat L., Hicks A. (2023). Impacts of Wildfire Smoke and Air Pollution on a Pediatric Population with Asthma: A Population-Based Study. Int. J. Environ. Res. Public Health.

[15] Hu Y., Cheng J., Liu S., Tan J., Yan C., Yu G., Yin Y., Tong S. (2022). Evaluation of climate change adaptation measures for childhood asthma: A systematic review of epidemiological evidence. Sci. Total. Environ..

[16] Anenberg S.C., Mohegh A., Goldberg D.L., Kerr G.H., Brauer M., Burkart K., Hystad P., Larkin A., Wozniak S., Lamsal L. (2022). Long-term trends in urban NO_2_ concentrations and associated paediatric asthma incidence: Estimates from global datasets. Lancet Planet. Health.

[17] Pivniouk V., Gimenes Junior J.A., Honeker L.K., Vercelli D. (2020). The role of innate immunity in asthma development and protection: Lessons from the environment. Clin. Exp. Allergy.

[18] Hallit S., Sacre H., Kheir N., Hobeika E., Hallit R., Waked M., Salameh P. (2021). Hygiene hypothesis: Association between hygiene and asthma among preschool children in Lebanon. Allergol. Immunopathol..

[19] Alshahwani N., Briatico D., Lee W., Farrokhyar F. (2022). Review and Quality Assessment of Systematic Reviews and Meta-analyses on the Management of Pediatric Inguinal Hernias: A Descriptive Study. J. Surg. Res..

[20] Su Q., Gao S., Chen J., Lu C., Mao W., Wu X., Zhang L., Zuo L. (2020). A Comparative Study on the Clinical Efficacy of Modified Circumcision and Two Other Types of Circumcision. Urol. J..

[21] Fraser J.D., Duran Y.K., Deans K.J., Downard C.D., Fallat M.E., Gadepalli S.K., Hirschl R.B., Lal D.R., Landman M.P., Leys C.M. (2023). Natural history and consequence of patent processus vaginalis: An interim analysis from a multi-institutional prospective observational study. J. Pediatric Surg..

[22] Radacsi A., Katona K., Farkas N., Kovesi T., Szanto I., Sandor B. (2023). Pain-related complaints of paediatric patients after dental treatment under general anaesthesia. Eur. J. Paediatr. Dent..

[23] Gomez-Rios I., Perez-Silva A., Serna-Munoz C., Ibanez-Lopez F.J., Periago-Bayonas P.M., Ortiz-Ruiz A.J. (2023). Deep Sedation for Dental Care Management in Healthy and Special Health Care Needs Children: A Retrospective Study. Int. J. Environ. Res. Public Health.

[24] McInnis-Smith K., Chen K., Klanderman M., Abruzzo T., Ramasubramanian A. (2022). Quantity and duration of exposure to general anesthesia for pediatric patients with retinoblastoma. J. AAPOS.

[25] Kim K.H., Chung J.H., Byun H., Zheng T., Jeong J.H., Lee S.H. (2020). Clinical Characteristics of External Auditory Canal Foreign Bodies in Children and Adolescents. Ear. Nose Throat J..

[26] Awad A.H., ElTaher M. (2018). ENT Foreign Bodies: An Experience. Int. Arch. Otorhinolaryngol..

[27] Thung A., Tumin D., Uffman J.C., Tobias J.D., Buskirk T., Garrett W., Karczewski A., Saadat H. (2018). The Utility of the Modified Yale Preoperative Anxiety Scale for Predicting Success in Pediatric Patients Undergoing MRI Without the Use of Anesthesia. J. Am. Coll. Radiol..

[28] Oubenyahya H., Bouhabba N. (2019). General anesthesia in the management of early childhood caries: An overview. J. Dent. Anesth. Pain Med..

[29] Salaun J.P., Poirel N., Dahmani S., Chagnot A., Gakuba C., Ali C., Gerard J.L., Hanouz J.L., Orliaguet G., Vivien D. (2021). Preventing the Long-term Effects of General Anesthesia on the Developing Brain: How Translational Research can Contribute. Neuroscience.

[30] Yang Y.L., Wang L.J., Chang J.C., Ho S.C., Kuo H.C. (2021). A National Population Cohort Study Showed That Exposure to General Anesthesia in Early Childhood Is Associated with an Increase in the Risk of Developmental Delay. Children.

[31] Salaun J.P., Chagnot A., Cachia A., Poirel N., Datin-Dorriere V., Dujarrier C., Lemarchand E., Rolland M., Delalande L., Gressens P. (2023). Consequences of General Anesthesia in Infancy on Behavior and Brain Structure. Anesth. Analg..

[32] Chen Y.R., Zhang S.X., Fang M., Zhang P., Zhou Y.F., Yu X., Zhang X.N., Chen G. (2022). Egr2 contributes to age-dependent vulnerability to sevoflurane-induced cognitive deficits in mice. Acta Pharmacol. Sin..

[33] Ing C., Bellinger D.C. (2022). Long-term cognitive and behavioral outcomes following early exposure to general anesthetics. Curr. Opin. Anaesthesiol..

[34] Kuo H.C., Yang Y.L., Ho S.C., Guo M.M., Jiang J.H., Huang Y.H. (2016). General anesthesia exposure in early life reduces the risk of allergic diseases: A nationwide population-based cohort study. Medicine.

[35] Goretzki A., Zimmermann J., Rainer H., Lin Y.J., Schulke S. (2022). Immune Metabolism in TH2 Responses: New Opportunities to Improve Allergy Treatment—Disease-Specific Findings (Part 1). Curr. Allergy Asthma Rep..

[36] Feng M., Feng Q., Chen Y., Liu G., Gao Z., Xiao J., Feng C. (2021). Effect of Dezocine on the Ratio of Th1/Th2 Cytokines in Patients Receiving Postoperative Analgesia Following Laparoscopic Radical Gastrectomy: A Prospective Randomised Study. Drug Des. Dev. Ther..

[37] Sheeran P., Hall G.M. (1997). Cytokines in anaesthesia. Br. J. Anaesth..

[38] Sofra M., Fei P.C., Fabrizi L., Marcelli M.E., Claroni C., Gallucci M., Ensoli F., Forastiere E. (2013). Immunomodulatory effects of total intravenous and balanced inhalation anesthesia in patients with bladder cancer undergoing elective radical cystectomy: Preliminary results. J. Exp. Clin. Cancer Res. CR.

[39] Cheng T.M. (2015). Reflections on the 20th anniversary of Taiwan's single-payer National Health Insurance System. Health Aff..

[40] Kuo H.C., Chang W.C., Yang K.D., Yu H.R., Wang C.L., Ho S.C., Yang C.Y. (2013). Kawasaki disease and subsequent risk of allergic diseases: A population-based matched cohort study. BMC Pediatr..

[41] Chang W.P., Wu S.J., Chang W.C., Kuo H.C. (2013). Population-based study of the association between urbanization and Kawasaki disease in Taiwan. Sci. World J..

[42] Burgmeier C., Dreyhaupt J., Schier F. (2015). Gender-related differences of inguinal hernia and asymptomatic patent processus vaginalis in term and preterm infants. J. Pediatric Surg..

[43] Chauhan B.F., Chartrand C., Ni Chroinin M., Milan S.J., Ducharme F.M. (2015). Addition of long-acting beta2-agonists to inhaled corticosteroids for chronic asthma in children. Cochrane Database Syst. Rev..

[44] Rehder K.J. (2017). Adjunct Therapies for Refractory Status Asthmaticus in Children. Respir. Care.

[45] Carrie S., Anderson T.A. (2015). Volatile anesthetics for status asthmaticus in pediatric patients: A comprehensive review and case series. Paediatr. Anaesth..

[46] Shankar V., Churchwell K.B., Deshpande J.K. (2006). Isoflurane therapy for severe refractory status asthmaticus in children. Intensive Care Med..

[47] Wasowicz M., Jerath A. (2014). Expanding the use of volatile anesthetic agents beyond the operating room. Can. J. Anaesth..

[48] Vaschetto R., Bellotti E., Turucz E., Gregoretti C., Corte F.D., Navalesi P. (2009). Inhalational anesthetics in acute severe asthma. Curr. Drug Targets.

[49] Burburan S.M., Xisto D.G., Rocco P.R. (2007). Anaesthetic management in asthma. Minerva Anestesiol..

[50] Bayable S.D., Melesse D.Y., Lema G.F., Ahmed S.A. (2021). Perioperative management of patients with asthma during elective surgery: A systematic review. Ann. Med. Surg..

[51] Kim D.C., Choi Y.W., Lee E.S., Choi J.W. (2022). No Association Between First Exposure to General Anaesthesia and Atopic Dermatitis in the Paediatric Population. Acta Derm. Venereol..

[52] Schneemilch C.E., Hachenberg T., Ansorge S., Ittenson A., Bank U. (2005). Effects of different anaesthetic agents on immune cell function in vitro. Eur. J. Anaesthesiol..

[53] Duffen A., Williams A. (2011). Should sevoflurane be used for maintenance of anaesthesia in children?. Br. J. Hosp. Med..

[54] Palacios A., Mencia S., Llorente A.M., Cruz J., Toledo B., Ordonez O., Olmedilla M., Lopez-Herce J. (2016). Sevoflurane Therapy for Severe Refractory Bronchospasm in Children. Pediatr. Crit. Care Med..

[55] Costi D., Cyna A.M., Ahmed S., Stephens K., Strickland P., Ellwood J., Larsson J.N., Chooi C., Burgoyne L.L., Middleton P. (2014). Effects of sevoflurane versus other general anaesthesia on emergence agitation in children. Cochrane Database Syst. Rev..

[56] Regli A., Sommerfield A., von Ungern-Sternberg B.S. (2022). Anesthetic considerations in children with asthma. Paediatr. Anaesth..

[57] Cheng Y., Wang J., Wu N., Zhang L., Jiang H. (2020). Multiple sevoflurane exposures don’t disturb the T-cell receptor repertoire in infant rhesus monkeys’ thymus. Life Sci..

[58] Chutipongtanate A., Prukviwat S., Pongsakul N., Srisala S., Kamanee N., Arpornsujaritkun N., Gesprasert G., Apiwattanakul N., Hongeng S., Ittichaikulthol W. (2020). Effects of Desflurane and Sevoflurane anesthesia on regulatory T cells in patients undergoing living donor kidney transplantation: A randomized intervention trial. BMC Anesthesiol..

[59] Elena G., Amerio N., Ferrero P., Bay M.L., Valenti J., Colucci D., Puig N.R. (2003). Effects of repetitive sevoflurane anaesthesia on immune response, select biochemical parameters and organ histology in mice. Lab. Anim..

[60] Gerber T.J., Fehr V.C.O., Oliveira S.D.S., Hu G., Dull R., Bonini M.G., Beck-Schimmer B., Minshall R.D. (2019). Sevoflurane Promotes Bactericidal Properties of Macrophages through Enhanced Inducible Nitric Oxide Synthase Expression in Male Mice. Anesthesiology.

[61] Wang L., Zha B., Shen Q., Zou H., Cheng C., Wu H., Liu R. (2018). Sevoflurane Inhibits the Th2 Response and NLRP3 Expression in Murine Allergic Airway Inflammation. J. Immunol. Res..

[62] Ju L.S., Jia M., Sun J., Sun X.R., Zhang H., Ji M.H., Yang J.J., Wang Z.Y. (2016). Hypermethylation of Hippocampal Synaptic Plasticity-Related genes is Involved in Neonatal Sevoflurane Exposure-Induced Cognitive Impairments in Rats. Neurotox. Res..

[63] Mori K., Iijima N., Higo S., Aikawa S., Matsuo I., Takumi K., Sakamoto A., Ozawa H. (2014). Epigenetic suppression of mouse Per2 expression in the suprachiasmatic nucleus by the inhalational anesthetic, sevoflurane. PLoS ONE.

[64] Yu H., Chen L., Yue C.J., Xu H., Cheng J., Cornett E.M., Kaye A.D., Urits I., Viswanath O., Liu H. (2022). Effects of propofol and sevoflurane on T-cell immune function and Th cell differentiation in children with SMPP undergoing fibreoptic bronchoscopy. Ann. Med..

[65] Ackerman R.S., Luddy K.A., Icard B.E., Pineiro Fernandez J., Gatenby R.A., Muncey A.R. (2021). The Effects of Anesthetics and Perioperative Medications on Immune Function: A Narrative Review. Anesth. Analg..

[66] Helmy S.A., Wahby M.A., El-Nawaway M. (1999). The effect of anaesthesia and surgery on plasma cytokine production. Anaesthesia.

